# *Kalrn* plays key roles within and outside of the nervous system

**DOI:** 10.1186/1471-2202-13-136

**Published:** 2012-11-01

**Authors:** Prashant Mandela, Maya Yankova, Lisa H Conti, Xin-Ming Ma, James Grady, Betty A Eipper, Richard E Mains

**Affiliations:** 1 Department of Neuroscience, University of Connecticut Health Science Center, Farmington, CT, 06030-3401, USA; 2 Molecular Microbial & Structural Biology, University of Connecticut Health Science Center, Farmington, CT, 06030-3401, USA; 3 Psychiatry, University of Connecticut Health Science Center, Farmington, CT, 06030-3401, USA; 4 Connecticut Institute Clinical Translational Science, University of Connecticut Health Science Center, Farmington, CT, 06030-3401, USA

**Keywords:** Neuromuscular junction, Rotarod, Grip strength, Anxiety, Passive avoidance, Rho-GEF

## Abstract

**Background:**

The human *KALRN* gene, which encodes a complex, multifunctional Rho GDP/GTP exchange factor, has been linked to cardiovascular disease, psychiatric disorders and neurodegeneration. Examination of existing *Kalrn* knockout mouse models has focused only on neuronal phenotypes. However, Kalirin was first identified through its interaction with an enzyme involved in the synthesis and secretion of multiple bioactive peptides, and studies in *C.elegans* revealed roles for its orthologue in neurosecretion.

**Results:**

We used a broad array of tests to evaluate the effects of ablating a single exon in the spectrin repeat region of *Kalrn* (KalSR^KO/KO^); transcripts encoding *Kalrn* isoforms containing only the second GEF domain can still be produced from the single remaining functional *Kalrn* promoter. As expected, KalSR^KO/KO^ mice showed a decrease in anxiety-like behavior and a passive avoidance deficit. No changes were observed in prepulse inhibition of acoustic startle or tests of depression-like behavior. Growth rate, parturition and pituitary secretion of growth hormone and prolactin were deficient in the KalSR^KO/KO^ mice. Based on the fact that a subset of *Kalrn* isoforms is expressed in mouse skeletal muscle and the observation that muscle function in *C.elegans* requires its *Kalrn* orthologue, KalSR^KO/KO^ mice were evaluated in the rotarod and wire hang tests. KalSR^KO/KO^ mice showed a profound decrease in neuromuscular function, with deficits apparent in KalSR^+/KO^ mice; these deficits were not as marked when loss of *Kalrn* expression was restricted to the nervous system. Pre- and postsynaptic deficits in the neuromuscular junction were observed, along with alterations in sarcomere length.

**Conclusions:**

Many of the widespread and diverse deficits observed both within and outside of the nervous system when expression of *Kalrn* is eliminated may reflect its role in secretory granule function and its expression outside of the nervous system.

## Background

Mammalian genomes encode 60–70 Rho GDP/GTP exchange factors (Rho-GEFs) and a similar number of Rho GTPase activating proteins (Rho-GAPs) to control the activation and inactivation of ~20 Rho-family GTPases [1]. The fact that mutations in individual Rho-GEFs are associated with specific disease phenotypes indicates that their functions are not redundant. For example, the *KALRN* gene, which encodes proteins with two Rho-GEF domains, has been associated with stroke [2], early onset coronary artery disease [2-5], schizophrenia [6-9] and adult attention deficit-hyperactivity disorder [10].

**Figure 1:**
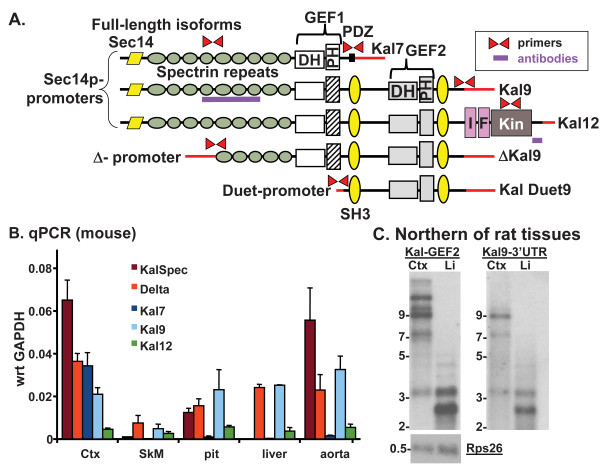
**Kalirin isoforms occur in many tissues.A**. The major isoforms of Kalirin are diagramed. The qPCR primers used to quantify expression of different regions of the Kalirin gene are indicated and antibody specificities are indicated. DH, Dbl homology; PH, Pleckstrin Homology; GEF1, Guanine nucleotide exchange factor 1; SH3, Src homology domain; GEF2, Guanine nucleotide exchange factor 2; I, Immunoglobulin; F, Fibronectin III; Kin, Kinase domain; red lines, unique 5^′^- and 3^′^-untranslated regions. **B**. Quantitative PCR was used to determine expression of different regions of the Kalirin gene. Data are from individual preparations of RNA and cDNA from 4–8 mice for each tissue. The data were averaged across assays, typically 4 assays per tissue. **C**. PolyA-containing RNA was prepared from adult rat cortex and liver, and hybridized with ^32^P]-labeled probes for the GEF2 domain (1023 nt) or the region immediately 3^′^ to the Kal9 stop codon (530 nt) [11]. Rps26 is ribosomal protein S26, used as a loading control.

The mouse *Kalrn* gene includes multiple promoters and several 3^′^-untranslated regions which produce functionally distinct isoforms in a tissue-specific and developmentally regulated manner [11-13]. While some Rho-GEFs consist of little more than the catalytic Dbl-homology (DH) domain followed by a pleckstrin homology (PH) domain, Kalirin is a complex protein with multiple catalytic, protein/protein and protein/lipid interaction domains. The longest isoform, Kalirin12, contains a lipid-binding Sec14 domain, nine spectrin-like repeats, two active Rho-GEF domains, two SH3 domains, an Ig/FnIII domain, and a kinase domain [14] (Figure 1). The most abundant isoform in the adult brain, Kalirin7, is almost exclusively localized to the postsynaptic density (PSD) [13,15], and plays an essential role in dendritic spine formation and function [16-19]. Kalirin9 and Kalirin12 are more highly expressed during nervous system development [20] and are also expressed in heart, skeletal muscle and endocrine tissue [20,21]. The single *Kalrn* orthologue in *C.elegans* (*UNC-73*) and *Drosophila* (*dTrio*) plays essential roles both within and outside of the nervous system [22,23].

Multiple promoters and 3^′^-terminal exons in the *Kalrn* gene preclude elimination of all isoforms through deletion of any one region of the gene. A knockout mouse for the most prevalent form of Kalirin in the adult brain, Kalirin7, was generated by eliminating its unique 3^′^-exon (Kal7^KO/KO^) [13]. Kal7^KO/KO^ mice have fewer dendritic spines in selected brain regions and exhibit impaired passive avoidance behavior, decreased anxiety-like behavior and accentuated locomotor sensitization to repeated cocaine treatment [13,24]. Cahill et al. [25] replaced *Kalrn* exons 27 and 28, which encode part of the first GEF domain, with the neomycin resistance gene, generating the KalGEF1^KO/KO^ mouse [25].

In this study, we flanked exon 13 in the spectrin repeat region with Lox-p sites (KalSR^CKO/CKO^); excision of this region makes it impossible to regain an in-reading-frame protein until exon 28. Global excision yielded KalSR^KO/KO^ mice while breeding of KalSR^CKO/CKO^ mice to mice expressing Cre-recombinase under control of the Nestin promoter largely limited excision to the nervous system (KalSR^NesKO/NesKO^). Based on our identification of Kalirin through its interaction with a secretory granule enzyme, the association of *Kalrn* with cardiovascular and psychiatric disease and the roles of *UNC-73* in *C.elegans*, we searched for deficits caused by lack of *Kalrn* within and outside of the nervous system.

## Methods

### Creation of global and nervous system specific *Kalrn* knockout mice

**Figure 2:**
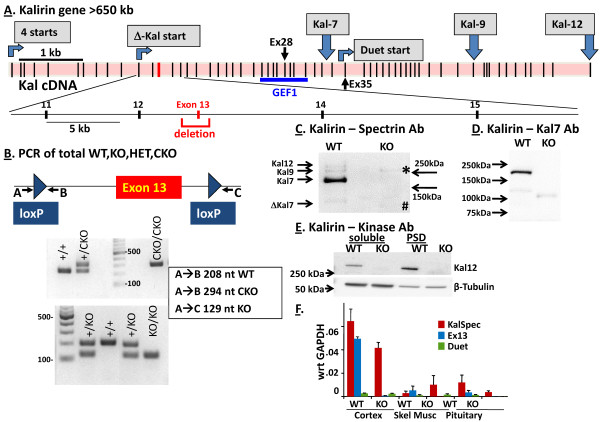
**Construction and verification of the Kalirin Exon 13 knockout (KalSR**^**KO/KO**^**). A**. Diagram of the mouse *Kalrn* gene with exons indicated as vertical bars; Exon 13 is shown in red. **B**. Detail of the knockout with PCR of DNA verifying the conditional and total knockouts. **C**. Kalirin proteins are absent from KalSR^KO/KO^ cortex. Kalirin was visualized using a Kalirin spectrin antibody; the band indicated by * was not detected by Kal12 antisera and is therefore considered non-specific. The band indicated by # is thought to represent an aberrant, N-terminally shortened product of the Δ-promoter. **D**. PSDs prepared from WT and KalSR^KO/KO^ cortex were analyzed using Kal7-specific antibody; the product that appears in the KalSR^KO/KO^ PSDs is thought to represent the protein encoded by Exon 13-lacking transcripts initiated in the Δ-promoter. **E**. Subcellular fractions probed using the Kal12-specific antibody showed the presence of Kalirin12 in the supernatant of a homogenate and in the PSD fraction in WT, but not KO, extracts. Tubulin was used as a loading control. **F**. Quantitative PCR was used to demonstrate the absence of transcripts containing Exon 13 in the KalSR^KO^ mouse, while transcripts containing the Duet start were still detected.

The basic strategy for ablating exon 13 was the same as for the Kalirin7-specific exon [13]. The Δ isoforms of Kalirin start at exon 11; exon 13 was chosen because exon 12 would have to be spliced to exon 28, in the middle of the GEF domain (Figure 2), to remain in the correct reading frame. Lox-p sites were introduced 1.6 kb upstream (nucleotide 34254054 on chromosome 16, mm9, July 2007) and 0.6 kb downstream of exon 13 (a 175 nt exon) (nucleotide 34251804). The strategy for removing the neomycin resistance cassette using flipper mice, breeding the conditional knockout mice into C57Bl/6 (Jackson Laboratories) and eliminating exon 13 using Hprt-Cre females was as described [13]. Mice with Lox-p sites flanking exon 13 are referred to as Kalirin Spectrin Repeat Conditional Knockout (KalSR^CKO^) mice; after Cre-mediated excision of exon 13, mice are referred to as Kalirin Spectrin Repeat Knockout (KalSR^KO^) mice. Both strains have been bred more than 10 generations into the C57Bl/6 background. KalSR^CKO/CKO^ and KalSR^+/CKO^ mice are of normal weight, reproduce well and have an unaltered distribution and level of Kalirin isoforms. KalSR^+/KO^ mice were bred to obtain KalSR^KO/KO^ mice. KalSR^CKO/CKO^ mice were crossed with C57Bl/6 mice expressing Cre recombinase under the control of the Nestin promoter [B6.Cg-Tg(Nes-cre)1Kln/J], to yield KalSR^NesKO^ mice; expression of Cre recombinase outside of the nervous and endocrine systems is minimal in this mouse, with restricted expression in the vasculature (http://jaxmice.jax.org/strain/003771.html).

### Behavioral studies

Animals were group housed in the Center for Comparative Medicine at the University of Connecticut Health Center (UCHC) with a 12 h light/dark cycle (lights on 7:00 am - 7:00 pm). All behavioral experiments were done in the Scoville Neurobehavioral Suite in accordance with UCHC Institutional Animal Care and Use Committee and National Institutes of Health guidelines. Male and female littermates 2 to 4 months of age were tested during the light phase. All animals were handled daily for at least 4 d before behavioral testing to minimize experimenter-induced stress. In addition, animals were allowed to habituate to the testing room for 1 h before testing. Animals were evaluated using several behavioral tests; the less stressful tests preceded those that were more stressful (open field→ elevated zero maze→ tail suspension test → prepulse inhibition of acoustic startle → rotarod→ grip test→ object recognition test→ passive avoidance test → restraint stress). Tests were separated by 2 or more days, and the same set of mice was used for all behavioral experiments. Open field, elevated zero maze, passive avoidance, and object recognition (male mice only) tests were performed as described [13]. Briefly, open field behavior was assessed by recording beam breaks for nine five-minute time bins using a San Diego Instruments clear Plexiglas chamber (38 cm × 38 cm). NaÃ¯ve mice were placed onto the elevated zero maze facing into the closed area and the time spent in the open area (all four feet) was monitored for 5 min. Passive avoidance behavior was monitored using a San Diego Instruments 2-chamber box; a 0.3 mA × 2 s scrambled footshock was delivered at the end of the training period, when the mouse was in the dark chamber. Novel object recognition was performed by placing the mouse into a clean rat cage containing two Falcon tubes or two LEGO^R^ objects and recording the time spent exploring each object over a three minute trial; the test was repeated the next day using the same object plus a novel object. The elevated zero maze and novel object recognition tests were videotaped and scored by a blinded observer. Data for male and female mice were analyzed separately; when no difference was observed, data were pooled.

#### Tail suspension

After taping the tail to a bent coat hanger, mice were suspended 70 cm above the floor of the cage for 6 min. Mice were videotaped and total time spent immobile (defined as the complete cessation of movement) was scored manually.

#### Rotarod

Training sessions (3 days, 3 trials per day) were conducted to acclimate the mice to the rotarod apparatus (Med Associates, VT). Motor coordination was assessed by measuring the length of time each mouse remained on the rotating rod as it accelerated from 4 to 40 rpm over a period of five min. On the 4th day, mice were tested in the same way three more times and the mean was used as the measure of competence at this task.

#### Wire hang

Two parallel steel wires 8 cm long and separated by 2 cm were anchored to the middle of a cardboard (40 cm  ×  22 cm); a 2 cm space separated the wires from the cardboard enabling a mouse to grip the wires with all four feet but not to climb onto the wires. The cardboard was suspended 30 cm above a rat cage containing bedding to prevent injury. Mice were trained for 3 days; on the fourth day, time spent suspended from the wires was measured. On the testing day, each mouse underwent 3 trials and the mean value was used to determine wire hang time.

#### Acute restraint stress

When behavioral testing was finished, mice were returned to their home cages for a week. Blood collected via submandibular puncture two days prior to restraint stress was used for measuring basal serum corticosterone levels. Restraint stress was performed between 10:00 AM and noon by placing mice into ventilated 50-ml plastic tubes for 15 minutes. Mice were then sacrificed by decapitation; serum prepared from trunk blood was stored at −80°C until assayed for corticosterone (MP Diagnostics) and peptidylglycine α-hydroxylating monoxygenase (PHM activity) [26].

#### Prepulse inhibition of the acoustic startle response

Male mice were subjected to acoustic startle testing and measurement of prepulse inhibition (PPI) using two identical startle chambers (San Diego Instruments) essentially as described for rats [27]. Mice were placed into a clear acrylic cylindrical chamber enclosed in a sound- and vibration-attenuating cabinet equipped with a 5 W bulb and ventilation fan. The chamber containing the mouse sits on a base that contacts a piezoelectric accelerometer that detects the whole body startle response. Output signals from the accelerometer are collected as 75 sequential 1 msec measurements starting at the onset of the startling stimulus (120 dB, 40 msec). Mice were allowed five min of acclimation prior to delivery of the stimuli over a 70 dB white noise background. The first and last six trials of the session consisted of the startle stimulus alone (120 dB, 40 ms). Trials occurred in a random order and consisted of 12 startle alone trials to calculate % PPI and 12 prepulse + startle trials at each of 5 prepulse intensities (3, 6,12,15,18 dB), and 8 no stimulus trials. Prepulse stimuli (20 ms) preceded startle stimuli by 100 ms. Accelerometer signals were rectified and digitized using SR-LAB (San Diego Instruments). Chambers were calibrated daily and matched for sound intensity [27].

#### Cardiovascular measurements

Blood pressure was measured using a non-invasive CODA tail-cuff blood pressure occlusion system (Kent Scientific). Measurements were taken in a quiet area away from other activity. Conscious animals were introduced into restraining chambers that were placed onto a preheated pad maintained at 30°C; tail temperature was measured using an infrared sensor and blood pressure measurements were initiated when tail temperature reached 30°C. Mice were trained for three days and on the fourth day BP recordings were made. Each measurement consisted of 5 acclimatization cycles followed by 15 blood pressure measurement cycles.

### Biochemical analyses

#### Analysis of DNA and RNA

DNA prepared from ear or tail snips [13] was used for genotyping with the primers shown in Figure 2B: A, TGTATGCCTTGGAAACAGGC; B, TGTTTTGCCATCGGGAGGAT; C, TGCAAGGAACATCGGGCTTT. The annealing temperature was 51°C (for 60 sec), elongation was at 72°C for 30 sec, repeated 42 times. Tissue RNA was prepared using Trizol as described [28]. Preparation of cDNA, quantitative polymerase chain reaction (qPCR) primers and conditions were as described for GAPDH, Kal7, Kal9, Kal12, Delta, and Kal-Spectrin [28]. New primers used in this study are shown in Table 1.

**Table 1:** Primers specific for Exon 13 and Duet

**Kalirin domain**	**Oligo name**	**Sequence**	**T**_**m**_**(°C)**	**Length (nt)**
Exon 13	Ex13-for	CTCAGCGATGTCCAACAACAAGACACC	61	121
	Ex13-rev	GAAGAGCTGTTTCACGAGCGGAAGATC	61	
Duet	Duet-for	CTGAAGTTTCCTACCGCCGCGC	60	122
	Duet-rev	AGCCCAAAGAGGGACCTCGGG	60	

#### Western blot analysis

Tissue fragments were solubilized in SDS lysis buffer [29] with protease inhibitors added at 10 μg/ml (benzamidine, leupeptin, soybean trypsin inhibitor, and aprotinin). After solubilization at 95°C, extracts were cleared of insoluble debris by centrifugation at 14,000 × *g*. Solubilized protein was quantified using a bicinchoninic acid assay with bovine serum albumin as the standard (Pierce). SDS-PAGE and immunoblotting with enhanced chemiluminescence were performed as described [13]. Rabbit antiserum JH2582 (raised to recombinant Kalirin spectrin repeats 4 to 7) and affinity-purified rabbit antiserum JH3225 (raised to the C-terminus of Kal12) [11] were visualized using horseradish peroxidase-conjugated anti-rabbit IgG.

### Secretion of growth hormone (GH) and prolactin (PRL)

Pools of adult male or female mouse pituitaries were used to prepare cultures as described [30]. Cells were plated at a density of 1.5 pituitaries per well in a protamine coated 96 well plate. Primary cells that had been maintained in culture for 2–3 days were pre-rinsed 2–3 times (15–30 min each) with secretion medium (complete serum-free medium [CSFM] containing 0.2 mg/ml BSA). To measure basal secretion cells were fed with secretion medium for 30 min and the fraction was collected. Following the basal collection, secretagogue stimulated secretion was initiated by adding medium containing 2 mM BaCl_2_ and/or 1 μM phorbol myristate acetate (PMA) for 30 min. Cells were harvested at the end of the secretion paradigm. Spent media (16% of sample) and cell lysates (1.6% of sample) were subjected to SDS-PAGE and western blot analysis using antibodies to GH (JH89) [31] or PRL (IC-5, National Hormone and Peptide Program [NIDDK, National Institutes of Health]); cell content of hormone was normalized to γ-adaptin (BD Transduction Laboratories).

### Visualization of neuromuscular junctions and muscle fine structure

#### Light microscopy

Nicotinic acetylcholine receptors (AChRs) were visualized in tibialis and diaphragm muscle using α-bungarotoxin-tetramethylrhodamine (Sigma-Aldrich, St. Louis, MO) as described [32]. Images were coded and neuromuscular junctions in tibialis muscle were categorized as normal (pretzel-shaped) or open (broken-pretzel). Our categorization was based on the criteria used to distinguish mature neuromuscular junctions from immature (plaque-shaped and partially differentiated) junctions [32]; each neuromuscular junction photographed *en face* was categorized as “normal” or “open” based on the number of discontinuities observed in its pretzel-like structure; we did not observe immature junctions in the adult tissue examined.

#### Electron microscopy

Animals were perfused transcardially with 4% paraformaldehyde (in 0.1 M sodium phosphate buffer, pH 7.4) under deep anesthesia with ketamine/xylazine. After fixation, the diaphragm was dissected out and placed into 4% paraformaldehyde in PBS for one hour. Diaphragm muscle was then incubated in PBS containing fluorescently-labeled α-bungarotoxin (100nM) at 4°C overnight. Neuromuscular junctions (NMJ) were identified under a fluorescence microscope and areas with NMJ’s were dissected out as 2mm cubes and processed for electron microscopy. The small cubes were further fixed with 2.5% glutaraldehyde in 0.1M sodium cacodylate buffer, pH 7.2 at 4°C. The following day, tissue was rinsed with cacodylate buffer and postfixed in 1% osmium tetroxide, 0.8% potassium ferricyanide in 0.1M cacodylate buffer for 1h at room temperature. Following standard procedures for dehydration, the tissues were embedded in Spurr resin. Ultrasections contrasted with uranylacetate were viewed on a Hitachi H-7650 transmission electron microscope and photographed. Coded images of neuromuscular junctions and muscle were scored by a blinded observer. Neuromuscular junctions with well defined boundaries containing neurotransmitter vesicles were considered for analysis. The number of neuromuscular junction folds per length of junction was measured using MetaMorph. The length of the sarcomere, A-band, I-band and Z-band was measured using MetaMorph. Data were obtained from three mice of each genotype.

### Statistical analyses

Data are presented as average ± SEM. Statistical analyses were performed with GraphPad Prism 4.0 (GraphPad Software, Inc., San Diego, CA, USA) or SPSS software. Data were analyzed using a t-test or one-way ANOVA with Bonferrroni correction as appropriate. Two-way repeated measures ANOVA was used to analyze the interaction between genotype and time in the open field or genotype and prepulse inhibition in the PPI test.

### Ethics statement

Consistent with granting approval, the respective UCHC committees (Institutional Animal Care and Use Committee; Safety) assessed the protocol to ensure the humane use and ethical care of vertebrate animals and the safety of employees. The UCHC Office of Compliance has rigorous policies and a well-established process for vetting actual or potential conflicts of interest. Prior to accepting a grant award, the Office of Research and Sponsored Programs, which serves as the institutional office responsible for submission of applications and administration of awards, coordinates and verifies that all proposed research has been approved by the committee having jurisdiction over the matter.

## Results

### Kalirin is expressed within and outside of the nervous system

Although most studies exploring Kalirin function have focused on its roles in the nervous system, its expression in skeletal muscle, liver and pituitary has been well documented [11,20,30,33]. We used several primer sets to compare *Kalrn* isoform expression in cortex, skeletal muscle, pituitary, liver and aorta in adult C57BL/6 mice using qPCR (Figure 1B). Cortex exhibited the expected pattern, with high expression of *Kalrn* isoforms that include all or part of the spectrin repeat region and the exon unique to Kalirin7. Expression of Kalirin7 was barely detectable outside of the nervous system.

Expression of *Kalrn* isoforms amplified by primers within the unique Kalirin9 3^′^-untranslated region (Figure 1B) or the GEF2 domain (not shown) was prominent in pituitary, liver and aorta, which also contained high levels of *Kalrn* transcripts that included all or part of the spectrin repeat region. Adult skeletal muscle expressed *Kalrn* transcripts amplified by Kalirin9 primers. Transcripts encoding *Kalrn* isoforms that could include the Sec14p domain (KalSpec primers) were notably absent from skeletal muscle and liver. Northern blot analysis using probes specific for GEF2 and Kalirin9 revealed the presence of 2.5 and 3.3 kb transcripts that could encode ΔKalirin9 and Kal Duet9 (Figure 1A) in liver polyA^+^ RNA (Figure 1C). The interaction of Kalirin with a secretory granule enzyme and the locomotor role of the *C.elegans* orthologue of *Kalrn* led us to include evaluations of pituitary and muscle function in our examination of the KalSR^KO/KO^ mouse.

### Generation and validation of KalSR^KO/KO^ mice

We targeted exon 13 of the *Kalrn* gene for elimination because splicing of exon 12 to any of the subsequent fifteen exons would yield an out-of-reading frame product. The strategy for eliminating exon 13 is outlined in Figure 2A [13]. As shown in Figure 2B, PCR analysis enabled monitoring of all of the expected genotypes from ear punches gathered when identifying weanlings. All PCR products were verified by DNA sequencing. KalSR^CKO/CKO^ mice were indistinguishable from wildtype C57BL/6 mice. Exon 13 was removed by breeding KalSR^CKO/+^ males with Hprt-Cre females; these mice lack 2.25 kb of chromosome 16 and retain one Lox-p sequence.

KalSR^KO/KO^ mice were generated by mating KalSR^+/KO^ mice and were viable. Western blot analysis using an antibody specific for the spectrin-repeat region of Kalirin confirmed the loss of Kalirin7, 9 and 12 from homogenates of adult cortex (Figure 2C). A cross-reactive protein slightly smaller than ΔKalirin7 was detected in the KalSR^KO/KO^ sample (Figure 2C; #). To determine its identity, transcripts initiated from the Δ-promoter were cloned and sequenced using cortical mRNA from KalSR^+/KO^ mice. Based on this analysis, the band indicated by # could be encoded by transcripts initiated from the Δ-promoter and lacking exon 13. Stop codons follow the first 5 AUG (start) codons in these transcripts but a 107 kDa protein lacking the first 139 residues of ΔKalirin7, replaced by 67 residues in a different reading frame, could be produced by translation that started at the sixth AUG. To test this hypothesis, a Kalirin7-specific antibody was used to analyze PSDs prepared from wildtype and KalSR^KO/KO^ cortex (Figure 2D); proteins the size of Kalirin7 and ΔKalirin7 were absent from the KalSR^KO/KO^ samples and a single product of the predicted mass was apparent. Use of an antibody specific for the region unique to Kalirin12 confirmed its loss from both the soluble and PSD-enriched fractions of adult cortex (Figure 2E).

We used qPCR to verify the elimination of transcripts containing *Kalrn* exon 13 and to evaluate expression of transcripts initiated at the *Kalrn* Duet promoter, which is located in the intron preceding exon 35 (Figure 2A). As expected, exon 13-containing transcripts were eliminated in all tissues examined (Figure 2F). Transcripts initiated at the Duet promoter were not eliminated and were present in cortex, skeletal muscle and pituitary. KalSR^KO/KO^ mice lack *Kalrn* transcripts initiated at five of the six major *Kalrn* promoters. The consensus for nonsense-mediated decay is that mRNAs are targeted for destruction when translating ribosomes encounter a stop codon followed within a short distance by a downstream splice site [34-36]. For Kalirin transcripts initiated at the full-length Sec14-promoters, the first stop codon produced by splicing exon 12 to exon 14 is not followed closely by a splice site; since these transcripts might not be subject to nonsense-mediated decay, it was expected that qPCR for the Kalirin Spectrin region (from Exon 10 to Exon 11) could yield normal levels in KalSR^KO/KO^ mice.

### KalSR^KO/KO^ mice show deficits at parturition

**Figure 3:**
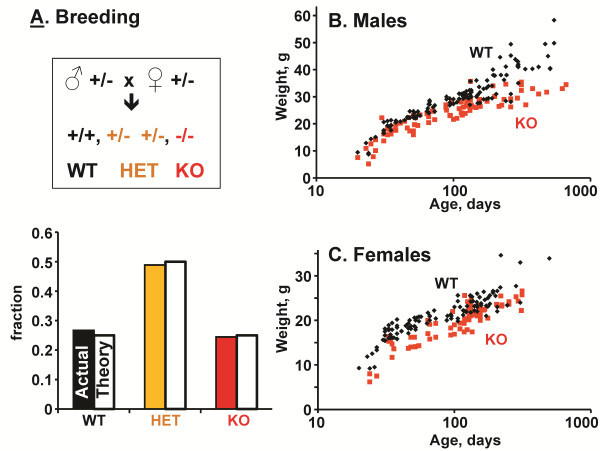
**Kalirin Exon 13 knockout results in growth retardation.A**. After breeding into a C57BL/6 background, matings of KalSR^KO/+^ males and females yielded WT, KalSR^KO/+^ and KalSR^KO/KO^ progeny in a Mendelian ratio. **B** and **C**. After weaning, WT and KalSR^KO/KO^ mice were weighed at regular intervals. Male (B) KalSR^KO/KO^ mice grew more slowly than WT mice. Female (C) KalSR^KO/KO^ mice were smaller at weaning but grew at the same rate as WT mice. Neither male nor female KalSR^KO/KO^ mice grew in a manner identical to WT mice (p<0.0002).

Once bred more than 10 generations into the C57Bl/6 background, the yield of pups from KalSR^+/KO^ matings matched the expected Mendelian 1:2:1 ratio (Figure 3A). Earlier in the process of breeding from a mixed 129/C57 background, the yield of KalSR^KO/KO^ pups was lower, as also seen with the Kalirin7^KO/KO^ mice [13]. Although female KalSR^KO/KO^ mice carried litters to term, most could not give birth and died during parturition, in spite of oxytocin administration. KalSR^KO/KO^ dams that gave birth showed deficits in nesting, pup rearing and milk letdown.

Weight data for WT and KalSR^KO/KO^ littermates were collected from weaning up to 2 years of age (Figure 3 B,C). A repeated measures general linear mixed model (GLMM) was used to analyze the growth curves using the MIXED procedure in SAS (SAS Institute Inc, Cary, NC) [37]. In these models the unit of analysis is the mouse and each mouse contributes a set of weights over time. A compound symmetry covariance structure was fit. We tested for equality of intercepts (weight at weaning) and slopes (growth rate) for the WT and KalSR^KO/KO^ mice. Male KalSR^KO/KO^ mice were not significantly smaller than WT mice at weaning (p=0.15) but grew at a slower rate, never attaining the normal growth curve (p<0.0002) (Figure 3B). Female KalSR^KO/KO^ mice were smaller than WT mice at weaning (p=0.003), grew at a similar rate (p=0.81) but never attained the normal growth curve (p<0.0002) [37](Figure 3C).

### Cultured pituitary cells reveal cell autonomous deficits in basal secretion in KalSR^KO/KO^

**Figure 4:**
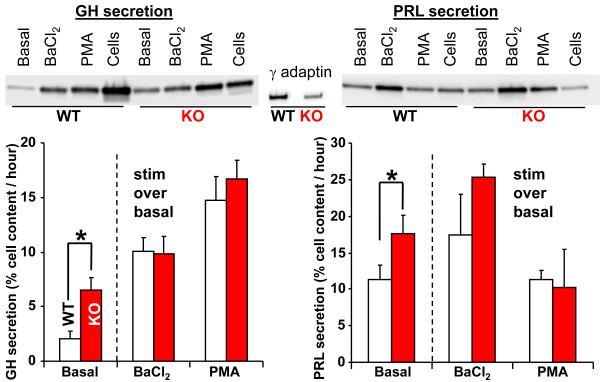
**KalSR**^**KO/KO **^**anterior pituitary cultures showed elevated basal secretion of growth hormone and prolactin.** Cultured anterior pituitary cells from male or female WT and KalSR^KO/KO^ mice were fed with basal medium followed by medium containing 2 mM BaCl_2_ , basal medium and medium containing 1 μM PMA. Cell lysates (1.67% of the total) and media (16.7% of the total) were probed by immunoblotting for growth hormone and prolactin; representative blots are shown. Secretion rates were calculated by expressing the amount of hormone in the medium as a percentage of the total amount of hormone in the cells at the end of the experiment; for the BaCl_2_ and PMA samples, the basal rate of secretion was subtracted from the total rate of secretion in the presence of secretagogue. Since no sex-specific differences in secretion rates were observed, data for male and female cultures were averaged. Basal growth hormone and prolactin secretion were increased in KalSR^KO/KO^ mice (growth hormone t(4)= 3.26, p<0.05; prolactin t(6)=4, p<0.05); *error bars* are S.E.M.

Kalirin is known to play a pivotal role in secretory granule maturation and storage [30]. Given these previous findings and the deficits in growth (Figure 3) and lactation, we used primary cultures to examine pituitary hormone secretion in KalSR^KO/KO^ mice. Although anterior pituitary somatotropes from KalSR^KO/KO^ mice synthesized and stored growth hormone normally, basal secretion of growth hormone (expressed as the percentage of cell content secreted per hour) was elevated compared to WT (t-test, p<0.05). In contrast, stimulation of secretion over basal levels by BaCl_2_ or phorbol myristate acetate was unaltered (Figure 4). Normal growth depends on pulsatile release of growth hormone [38], which reflects both hypothalamic input and nutrient feedback; any disruption in the normal pattern could contribute to deficient growth.

Prolactin secretion showed a similar genotypic difference, with increased basal secretion by KalSR^KO/KO^ lactotropes; since lactotropes *in vivo* are subject to tonic inhibition by dopamine, basal secretion of PRL in cell culture is substantially higher than basal secretion of GH. KalSR^KO/KO^ and wildtype lactotropes did not differ in their ability to increase prolactin secretion over basal levels in response to BaCl_2_ or PMA (Figure 4).

### Similarities in blood pressure, ability to respond to stress and basal locomotor activity in WT and KalSR^KO/KO^ mice

**Figure 5:**
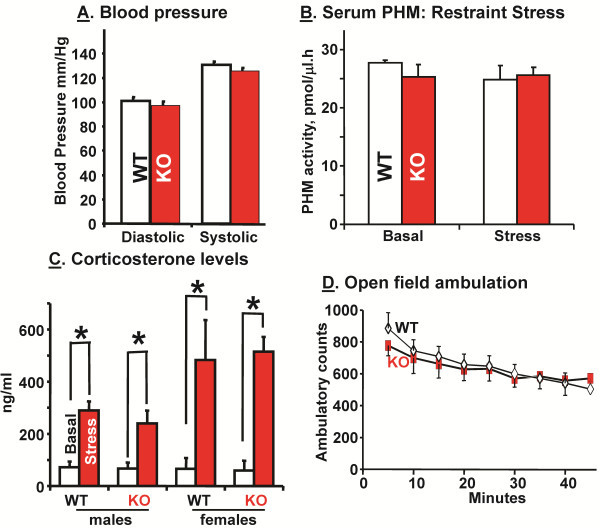
**Physiological and behavioral similarities between KalSR**^**KO/KO **^**and WT mice. A**. Blood pressure was measured in conscious animals using the tail-cuff method (CODA-2, Kent Scientific, Torrington, CT). Mice were trained for three days and on the fourth day BP recordings were made. Each measurement consisted of 5 acclimatization cycles followed by 15 BP measurements cycles; data for male and female mice did not differ and were pooled. There were no significant differences in the BP of WT and KalSR^KO/KO^ mice; *error bars* are S.E.M. **B**. Sera from male and female mice collected under basal conditions or after restraint stress (15 min) were subjected to PHM assay. Data for males and females did not differ and no significant differences were found between WT and KalSR^KO/KO^ mice; *error bars* are S.E.M. **C**. Serum corticosterone levels were measured in male and female mice under basal conditions and after a 15 min restraint stress; data for male and female mice are shown separately. No significant differences were found between genotypes; *error bars* are S.E.M. **D**. In the open field, WT and KalSR^KO/KO^ mice (males and females behaved similarly) showed time dependent decreases in spontaneous locomotor activity (F(8,208)=32.91 p<0.001), with no significant difference between genotypes over the 45 min test.

Since Kalirin has been implicated in early onset cardiovascular disease [39], resting blood pressure was evaluated in WT and KalSR^KO/KO^ mice; no significant differences were observed in this parameter, but Fanaroff et al. [5] have reported a role for *Kalrn* in smooth muscle cell migration/proliferation and atherogenesis (Figure 5A).

Our earlier work on the role of *Kalrn* in pituitary cells, deficits in peptidergic secretion in *C. elegans* expressing UNC-73 mutants and deficits in basal growth hormone and prolactin secretion by pituitary cells lacking *Kalrn* suggested that there might be widespread alterations in peptidergic secretion in KalSR^KO/KO^ mice. To evaluate this possibility, serum levels of a widely expressed secretory granule enzyme were monitored (Figure 5B); serum PHM activity was identical in WT and KalSR^KO/KO^ mice. As another test of peptidergic function, restraint stress was used to stimulate the hypothalamic-pituitary-adrenal axis in KalSR^KO/KO^ mice (Figure 5C); secretion of both hypothalamic corticotropin releasing hormone and pituitary adrenocorticotropic hormone is required for a normal response. Neither basal levels of corticosterone nor the ability to secrete corticosterone in response to stress was altered in male or female KalSR^KO/KO^ mice; as expected, corticosterone levels rose higher in female than in male mice after restraint stress (Figure 5C) [40].

Since many of the behavioral tests to be conducted rely on locomotor activity, we evaluated spontaneous locomotor activity in an open field chamber for 45 min; there were no significant differences between WT and KalSR^KO/KO^ mice (Figure 5D).

### Behavioral analysis of KalSR^KO/KO^ mice

**Figure 6:**
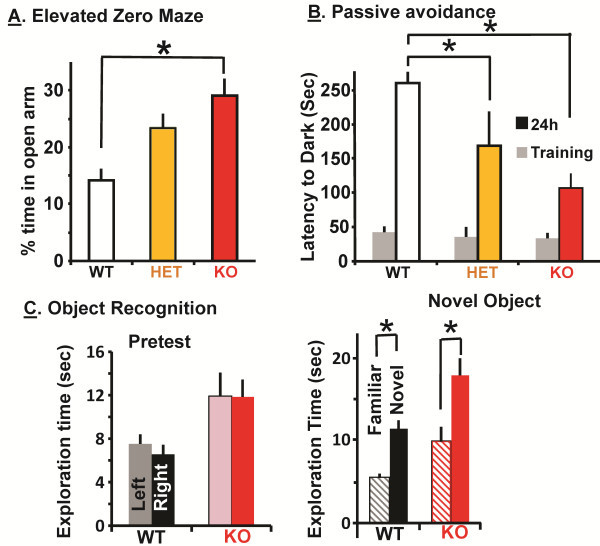
**KalSR**^**KO/KO **^**mice exhibit several behavioral changes similar to Kal7**^**KO/KO **^**mice. A**. Naive mice were subjected to the elevated zero maze test for 5 min. KalSR^KO/KO^ mice spent more time in the open arm than WT mice (F(2,35)=9.926, p<0.001); *error bars* are S.E.M. **B**. Genotype dependent impairment was evident in the passive avoidance test, with the greatest effect on KalSR^KO/KO^ mice (F(2,42)= 9.658; p<0.001). Bonferroni corrected t-tests revealed a difference between WT and KalSR^KO/+^ (p<0.05) mice but not between KalSR^+/KO^ and KalSR^KO/KO^ mice. **C**. A novel object recognition paradigm was used to determine learning and memory impairments in the KalSR^KO/KO^ mice. Both WT and KalSR^KO/KO^ mice spent significantly more time with the novel object (WT, t(28)=8, p<0.001)(KO, t(28)=5.5, p<0.001), but KalSR^KO/KO^ mice showed increased exploratory behavior compared to WT mice (t(28)=4.519,p<0.05 ); *error bars* are S.E.M. Male and female mice responded similarly in elevated zero maze and passive avoidance tests and data were pooled; the novel object recognition test was carried out only on male mice.

*KALRN* has been genetically associated with several neuropsychiatric disorders; therefore a battery of behavioral tests was carried out on WT and KalSR^KO/KO^ mice. Since Kalirin7 is also absent in KalSR^KO/KO^ mice, we first used the tests that revealed deficits in the Kal7^KO/KO^ mice. WT, KalSR^KO/+^ and KalSR^KO/KO^ mice were evaluated in the elevated zero maze; there was a clear effect of genotype on time spent in the open area, indicating a decrease in anxiety-like behavior (Figure 6A). KalSR^KO/KO^ mice spent a significantly longer time in the open area of the elevated zero maze than WT mice (Figure 6A). KalSR^+/KO^ mice did not differ significantly from WT or KalSR^KO/KO^ mice (Figure 6A).

Since *KALRN* transcript and Kalirin protein levels were reduced in hippocampi collected from Alzheimers patients [41], we wanted to test KalSR^KO/KO^ mice in hippocampal dependent tasks. Passive avoidance conditioning tests hippocampus-dependent memory [13]. During the training session, WT, KalSR^+/KO^ and KalSR^KO/KO^ mice showed the same latency to cross to the dark side of the chamber (Figure 6B). When tested 24 h after receiving a single foot shock, it was clear that genotype had a significant effect on the response. KalSR^KO/KO^ mice exhibited a significantly decreased latency to cross to the foot shock-paired side relative to WT mice. Once again, the KalSR^+/KO^ mice exhibited a response intermediate to that of WT and KalSR^KO/KO^ mice.

Kal7^KO/KO^ mice performed normally in the radial arm maze and in tests of novel object recognition [13]. KalSR^KO/KO^ mice spent significantly more time exploring both objects on the pretest day than did WT mice (Figure 6C). The total time spent exploring objects was also significantly higher for KalSR^KO/KO^ mice on the test day, but KalSR^KO/KO^ mice recognized the novel object as well as WT mice (Figure 6C). The increased object exploration observed in KalSR^KO/KO^ mice may reflect their decreased anxiety-like behavior or may reflect impaired response habituation [42] (Figure 6A).

**Figure 7:**
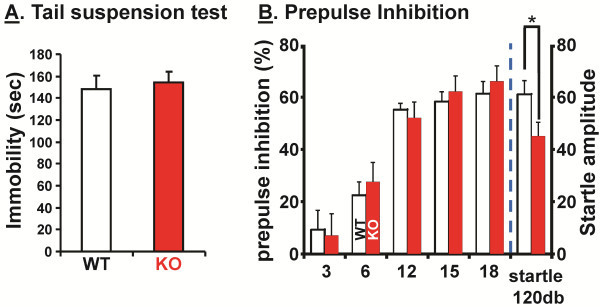
**KalSR**^**KO/KO **^**mice lack depressive-like symptoms and have normal sensorimotor gating.A **.WT and KalSR^KO/KO^ mice (male and female) showed no significant difference when subjected to tail suspension, to determine depressive-like behavior (t(16)=0.36; p>0.05). **B**. Behavioral similarities exist between WT and KalSR^KO/KO^ mice in the prepulse inhibition test; male mice of both genotypes showed a similar and significant prepulse inhibition (left y-axis) at all intensities (F (4,56)=61.27 p<0.001). In the pulse alone trials, KalSR^KO/KO^ mice showed a decreased startle response (right y-axis) compared to WT mice (t (14)=2.26; p<0.05); *error bars* are S.E.M.

Additional tests were used to evaluate the behavior of the KalSR^KO/KO^ mice. Depression-like behavior was evaluated using the tail suspension test (Figure 7A). There was no difference in immobility time for WT and KalSR^KO/KO^ mice.

Prepulse inhibition is the decrease in startle response to an intense auditory stimulus that is preceded by a smaller auditory prepulse [43]. Decreased *KALRN* mRNA levels were reported in the prefrontal cortex of subjects with schizophrenia [7,44], and PPI deficits due to altered sensory motor gating have been reported in schizophrenic patients and psychosis-prone individuals [45]. The startle response to pulse-alone trials in KalSR^KO/KO^ mice was significantly lower than in WT mice (Figure 7B). Contrary to our expectations, there was no significant difference between WT and KalSR^KO/KO^ mice in the prepulse induced inhibition of acoustic startle. However, the diminished startle response observed in the KalSR^KO/KO^ suggested a role for altered neuromuscular function, which was examined directly.

### KalSR^KO/KO^ and KalSR^+/KO^ mice show deficits in neuromuscular function

**Figure 8:**
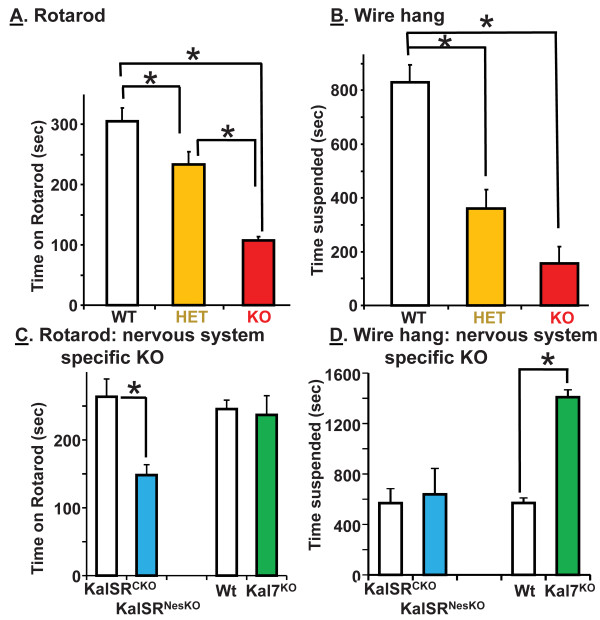
**KalSR**^**KO/KO **^**mice show loss of muscle strength, coordination and stamina.A**. After three training days, male and female mice were placed onto an accelerating rotarod, and time to fall from the rotarod was recorded for 3 successive trials separated by 20 min. KalSR^KO/KO^ mice stayed on the rotarod for less time than WT or Kal^+/KO^ mice (F(2,35)=39.68: p<0.0001). Bonferroni corrected t-tests revealed a significant difference between all genotypes. **B**. In the wire hang test, both KalSR^KO/KO^ and Kal^SR+/KO^ mice spent less time suspended than WT mice (F(2,32)=35.42, P<0.001). **C**. In the rotarod test, the performance of Kal^NesKO/NesKO^ mice was impaired compared to control KalSR^CKO/CKO^ mice (p<0.01). Kal7^KO/KO^ mice showed no impairment compared to WT controls. **D**. In the wire hang test, Kal^NesKO/NesKO^ mice and control KalSR^CKO/CKO^ mice spent equal amounts of time suspended. Kal7^KO/KO^ mice spent more time suspended than WT mice (p<0.01). Data for male and female mice were combined for the rotarod and wire hang tests.

Motor coordination was first tested using a rotarod; mice trained for three days were tested on the fourth day. Both the KalSR^+/KO^ and KalSR^KO/KO^ mice showed a decreased ability to perform this task relative to WT mice (Figure 8A); KalSR^KO/KO^ mice were more affected than KalSR^KO/+^ mice. Skeletal muscle function was further probed using a wire hang test. KalSR^+/KO^ and KalSR^KO/KO^ mice showed a drastic reduction in their ability to suspend themselves from a wire compared to WT mice (Figure 8B).

Since *Kalrn* is expressed in both motor neurons and skeletal muscle, the phenotype observed could result from the loss of *Kalrn* expression in either or both cell types. In order to direct elimination of *Kalrn* expression to the nervous system, KalSR^CKO/CKO^ mice were mated with nestin-Cre mice [46]. In these mice Cre is broadly expressed throughout the central nervous system from early embryonic development through adulthood; restricted expression is also observed in the vasculature and scattered cells in a wide variety of tissues (http://jaxmice.jax.org/strain/003771.html). KalSR^NesKO/NesKO^ and control KalSR^CKO/CKO^ mice were evaluated in the rotarod and wire hang tests. Since Kal7^KO/KO^ mice would be expected to mimic neuron-specific elimination of *Kalrn* expression, they were evaluated using the same tests. Compared to KalSR^KO/KO^ mice, KalSR^NesKO/NesKO^ mice exhibited less of a decrease in their ability to remain on the rotarod (Figure 8C); the performance of Kal7^KO/KO^ mice did not differ from that of WT mice in this test. In the wire hang test, KalSR^NesKO/NesKO^ mice were indistinguishable from the KalSR^CKO/CKO^ controls and did not exhibit the profoundly impaired performance observed in KalSR^KO/KO^ mice (Figure 8D); Kal7^KO/KO^ mice remained suspended from the wire for a longer time than WT mice.

### KalSR^KO/KO^ mice show deficits in neuromuscular junction and muscle structure

**Figure 9:**
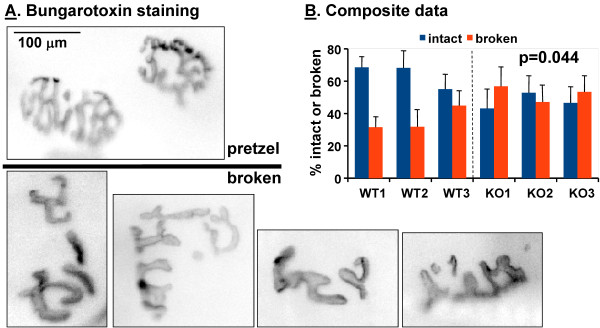
**Neuromuscular junctions are abnormal in KalSR**^**KO/KO **^**mice. A**.Individually teased fixed anterior tibialis muscles of adult WT and KalSR^KO/KO^ male and female mice were incubated with fluorescently tagged α-BTX to visualize postsynaptic AChR clusters. **B**. Neuromuscular junctions were categorized as normal (pretzel-shaped) or open (broken) and the percentage of the junctions categorized as normal was calculated for three WT and three KalSR^KO/KO^ mice; bars show data from a single mouse. KalSR^KO/KO^ mice have a lower fraction of intact pretzel shaped junctions (t-test; p=0.044) and a larger fraction of broken junction shapes (t-test; p=0.044).

In order to evaluate the structure of the neuromuscular junction, tibialis muscles from KalSR^KO/KO^ and WT mice were visualized after binding fluorescently tagged bungarotoxin to the nicotinic aceylcholine receptor (Figure 9A). The expected compact, pretzel-like structures were consistently observed in WT muscle while a more open, less well organized pattern was common in the KalSR^KO/KO^ muscle. Categorization using established criteria [32] suggested a significant difference in the organization of the nicotinic acetylcholine receptors at the neuromuscular junction in KalSR^KO/KO^ mice (Figure 9B).

**Figure 10:**
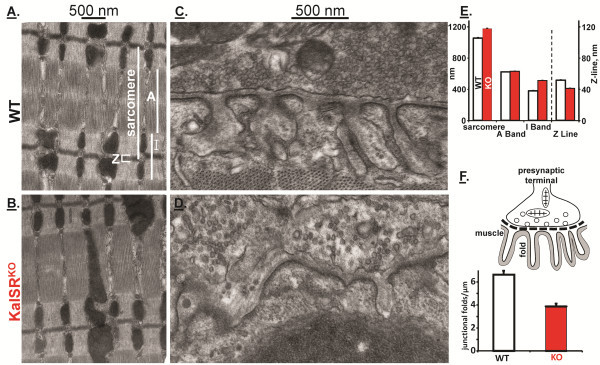
**KalSR**^**KO/KO **^**mice have abnormal skeletal muscle ultrastructure.** Diaphragm muscle from WT and KalSR^KO/KO^ male and female mice was prepared for electron microscopic analysis. Representative images showing alterations in muscle (**A,B**) and neuromuscular junction (**C,D**) architecture are shown. A sarcomere, with the A, I and Z bands marked, is shown in (**A**). **E**. KalSR^KO/KO^ mice showed increased sarcomere (t(4)=12,p<0.001), increased I-band (t(4)=19,p<0.001) and decreased Z-band (t(4)=12,p<0.01) length. **F**. Junctional folds, shown schematically, were quantified per μm of junctional membrane; KalSR^KO/KO^ mice showed a decrease in junctional fold density (t(4)=4 p<0.05).

For a more detailed, quantitative analysis, we turned to the diaphragm muscle; neuromuscular junctions were localized in fixed tissue and analyzed by transmission electron microscopy. In both WT and KalSR^KO/KO^ mice, muscle fibers were well organized (Figure 10A,B), with peripheral nuclei and normal mitochondrial content. Sarcomere length was increased in KalSR^KO/KO^ mice, reflecting an increase in I-band length; A-band length was unaltered (Figure 10E). Z-line thickness was decreased in KalSR^KO/KO^ muscle.

Neuromuscular junctions were identified based on the apposition of specialized regions of muscle membrane and nerve endings filled with synaptic vesicles and mitochondria [47]. Well organized junctional folds closely apposed to nerve terminals filled with synaptic vesicles were readily found in WT tissue (Figure 10C). In contrast, both the nerve terminals and junctional folds were disturbed in KalSR^KO/KO^ mice (Figure 10D). The number of junctional folds/μm of synaptic membrane was quantified by a blinded observer and was decreased in the KalSR^KO/KO^ mice (Figure 10F). In addition, mitochondria often accumulated in the presynaptic endings of KalSR^KO/KO^ mice. Differences in both neuronal and muscle tissue may contribute to the altered rotarod and wire hang performance.

## Discussion and conclusion

Genetic linkage studies and analyses of post-mortem tissue have associated human *KALRN* with a wide array of neuropsychiatric and cardiovascular diseases [6,7,10,24,25,39,48]. Detailed studies of the actions of Kalirin7, the major isoform in the adult nervous system, identified several pathways through which Kalirin7 can participate in spine formation and synaptic plasticity [13,17,18,49-51], but have not provided insight into the roles played by other Kalirin isoforms within or outside of the nervous system. Analysis of the *C. elegans* and *D. melanogaster* ortholog of *Kalrn* revealed essential roles for this gene both within and outside of the nervous system [52-55]; neither species has an isoform similar to Kalirin7.

### Functions unique to Kalirin9 and Kalirin12

Kalirin9 and Kalirin12 are expressed in skeletal muscle, pituitary, liver and aorta, but very little Kalirin7 is expressed outside of the nervous system. The second GEF domain of *Kalrn*, which is present in Kalirin9 and Kalirin12, but absent from Kalirin7, activates RhoA, not Rac1 or RhoG. The downstream effectors of Rac1 and RhoA are distinctly different [1,56,57] and many of the disease-associated mutations identified in human *KALRN* map near the second GEF domain [6]. In the fly and worm orthologs of *Kalrn*, the Rac- and Rho-specific GEF domains play different but essential roles [52-54]. This strongly suggests unique roles for the *Kalrn* isoforms that contain the Rho-specific GEF2 domain. The GEF2 domain of *UNC-73* plays a role in muscle contraction triggered through G protein coupled receptors signaling through Gα_q_[22]. Structural analysis of a Gα_q_-p63RhoGEF-RhoA complex indicates that Gα_q_ relieves the autoinhibitory effect of the PH domain; Gα_q_ also activates the GEF2 domain of *Kalrn*[58]. Interactions unique to Kalirin12 have also been identified [59]; the binding of dynamin to the Ig/Fn domain is consistent with a role for *Kalrn* in endocytosis [60]. The fly and worm orthologs of *Kalrn* lack a kinase domain and offer no insight into its potential roles.

By comparing the phenotypes of the different *Kalrn* knockout mouse lines, we identified deficits caused by the loss of all of the Kalirin isoforms that were not observed in mice lacking only Kalirin7. Deficits observed in the KalSR^KO/KO^ mice but not in the Kal7^KO/KO^ mice presumably reflect the functions of Kalirin9 and −12 both within and outside of the nervous system. Deficits common to KalSR^KO/KO^ and Kal7^KO/KO^ mice reflect sites at which Kalirin7 is expressed and its unique localization to the PSD, where it can interact with PDZ domain containing proteins.

### Deficits unique to KalSR^KO/KO^ mice

One of the most striking deficits unique to KalSR^KO/KO^ mice was their inability to perform in the rotarod and wire hang tests. That KalSR^+/KO^ heterozygote mice also failed to perform normally in either test suggests that motor deficits are a sensitive indicator of compromised *Kalrn* function. Examination of the neuromuscular junction in KalSR^KO/KO^ mice revealed deficits in both the muscle and the presynaptic ending. A role for *Kalrn* in muscle is consistent with findings in *C. elegans*[22]. Splice variants of *UNC-73* that resemble Kal Duet 9, a mammalian isoform generated from the Duet promoter, play essential roles in pharynx and vulval muscle [22]. In mammals, the Duet promoter also yields Kal Duet 12, which contains both a GEF domain and an active kinase domain [33].

The extensive signaling that occurs between skeletal muscle and the innervating motor neuron means that further experiments will be required to distinguish the roles of the different *Kalrn* isoforms in these two tissues. Kalirin9 and Kalirin12 are the most prevalent isoforms in the brain during embryonic development, and exogenous Kalirin9 or Kalirin12 in superior cervical ganglion neurons stimulated the initiation, growth and branching of axons [61]. Kalirin9 and Kalirin12 decline when Kalirin7 expression increases during the burst of synaptogenesis that occurs after birth [16]. Kalirin9, but not Kalirin7 or Kalirin12, binds p75 and regulates p75-Nogo receptor-dependent RhoA activation in cerebellar granule neurons in culture, inhibiting myelin formation [62]. In *Drosophila*, dTrio interacts with Notch, affecting axon sprouting and guidance [63]; the GEF1 domain, but not the GEF2 domain, is essential for this interaction [23]. *dTrio* expressed in neurons is also a key component of the BMP retrograde signaling pathway used to coordinate pre- and post-synaptic development of the neuromuscular junction [55].

KalSR^NesKO/NesKO^ mice, which lack all isoforms of *Kalrn* in the nervous system, exhibited a deficit in rotarod behavior but were not impaired to the same extent as KalSR^KO/KO^ mice; Kal7^KO/KO^ mice behaved like WT mice. Thus it is clear that isoforms of *Kalrn* expressed outside of the nervous system play an essential role in rotarod performance. Since Kalirin9 and Kalirin12 are expressed in skeletal muscle, the deficit may be a direct result of their absence from muscle cells. A better understanding of the role of Kalirin in skeletal muscle requires identifying the sites at which it is located. The clustering of receptors, ion channels and signaling proteins that occurs at the neuromuscular junction involves proteins similar to those involved in the formation of the PSD and skeletal muscle Kalirin could play a role in this process. The effect of Kalirin on the Z-line could be direct or indirect; many of the same proteins essential to spine formation and function participate in Z-line formation. The increase in sarcomere length observed in KalSR^KO/KO^ diaphragm muscle arose from an increase in the length of the I-band, which consists of actin filaments whose pointed ends extend between the myosin filaments. The barbed ends of the anti-parallel actin filaments from adjacent sarcomeres meet at the Z-line, which showed a decrease in width in KalSR^KO/KO^ mice. Z-line width is thought to correlate with the layers of α-actinin that cross-link the anti-parallel actin filaments [64]. α-Actinin, which binds to NMDA receptors, is enriched at the PSD; when over-expressed in hippocampal neurons, α-actinin increases the formation of long dendritic protrusions [65].

While loss of Kalirin expression in skeletal muscle may contribute to the inability of KalSR^KO/KO^ mice to perform in the wire hang test, it is clear that expression of Kalirin7 in the nervous system also plays an important role in this behavioral response. Kal7^KO/KO^ mice remained suspended from the wire for a far longer time than WT mice, suggesting that central control of skeletal muscle has been altered. The KalSR^NesKO/NesKO^ mice exhibit a response intermediate to that of KalSR^KO/KO^ and Kal7^KO/KO^ mice and similar to that of KalSR^CKO/CKO^ mice. The many pathways through which the CNS controls skeletal muscle contraction make it impossible to identify the sites at which Kalirin7 plays an essential role, but it is not uncommon for mouse mutants to show differential susceptibility to changes in performance in the wire hang and rotarod tests [66,67].

The novel object recognition test revealed an intriguing difference between KalSR^KO/KO^ and Kal7^KO/KO^ mice; while KalSR^KO/KO^ mice spent more total time exploring the objects than WT mice, Kal7^KO/KO^ mice did not. This difference suggests that Kalirin9 and/or Kalirin12 contribute to this behavior. The age dependent hyperactive behavior reported in KalGEF1^KO/KO^ mice [25] may be similar to the enhanced exploration of both familiar and novel objects seen with the KalSR^KO/KO^ mice, and may reflect the absence of Kalirin9 and/or Kalirin12. Due to the absence of Kalirin isoforms that include the GEF2 domain, the functioning of pathways that rely on GPCR signaling through Gα_q_ to RhoA may be impaired. Both cholinergic signaling through M1, M3 and M5 muscarinic receptors [68] and serotonergic signaling through 5HT_2A_ and 5HT_2C_ receptors involve Gα_q_[69,70].

A role for Kalirin in secretory granule release was expected from previous studies using pituitary cells in culture [30], from the fact that Kalirin was first identified through its interaction with a secretory granule membrane protein and from the altered neurotransmitter release observed in worms and flies [22,52-55]. The increased basal secretion of PRL and GH observed in KalSR^KO/KO^ pituitary cells in culture may underlie the growth deficits in the animal, since elevated GH without the normal periodicity in its secretion contributes to stunted growth in experimental animals and across the human population [38,71,72]. Altered basal secretion of peptides could be expected to contribute to other deficits observed in KalSR^KO/KO^ mice, especially at the neuromuscular junction.

### Deficits common to KalSR^KO/KO^ and Kal7^KO/KO^ mice

A similar decrease in baseline anxiety-like behavior was seen in both KalSR^KO/KO^ and Kal7^KO/KO^ mice. KalSR^KO/KO^ mice and Kal7^KO/KO^ mice also exhibited comparably impaired passive avoidance behavior. Since Kal7^KO/KO^ mice produce slightly increased amounts of Kalirin9 and Kalirin12 compared to WT mice [13], we think it likely that a lack of Kalirin7 causes the changes observed in the affected pathways. It is clear that Kalirin7 plays an essential role in the formation and function of excitatory synapses on dendritic spines. Both NMDA receptor dependent LTP and LTD are deficient in Kal7^KO/KO^ mice [73]. In the absence of Kalirin7, chronic cocaine treatment is unable to increase the size or the number of spines on the dendrites of medium spiny neurons in the nucleus accumbens [24]. In hippocampal cultures, a decrease in Kalirin7 expression both reduced the density of excitatory synapses and the ability of estadiol to increase synapse formation [19].

Expression of Kalirin in the amygdala, one of the regions that plays a critical role in anxiety-like behavior and passive avoidance, is not notably different from expression levels elsewhere in the brain, but region or cell-type specific differences in the expression of key interactors may determine which pathways are most sensitive to a decrease in Kalirin7 levels. Many of proteins identified in a screen for Kalirin7 PDZ binding motif interactors are known to be involved in the clustering of NMDA and AMPA receptors (PSD-95, SAP102, SAP97, Chapsyn-110, PICK-1) [15,49]. Other interactors link Kalirin7 to filamentous actin (ZO-1, ZO-2, neurabin, spinophilin, AF-6/afadin), a key determinant of spine shape and thus of spine function. Of special interest, given the role of serotoninergic transmission in anxiety and fear, is MUPP1, which binds to both Kalirin7 and to the 5HT_2A_ and 5HT_2C_ receptors [15,74].

Kalirin7, Kalirin9 and Kalirin12 share the ability to interact with a wide variety of proteins including peptidylglycine α-amidating monoxygenase (PAM) [75], DISC-1 [9], TrkB [76], inducible NOS [77], NR2B [29] and phosphoinositides [60] through their common Sec14, spectrin repeat and GEF1 domains.

### Manipulating *Kalrn* to study psychiatric/neurological disorders

*KALRN* has been linked to schizophrenia and adult ADHD, and mouse models for these diseases would be of great use. Patients suffering from a variety of neuropsychiatric disorders including schizophrenia, obsessive compulsive disorder and Huntington’s disease often exhibit deficits in prepulse inhibition [45,78,79]. The linkage of *KALRN t*o schizophrenia and the interaction of Kalirin with DISC-1 made prepulse inhibition, which can be modeled in rodents [45], of particular interest. However, in a test of prepulse inhibition of acoustic startle, KalSR^KO/KO^ mice showed no sensorimotor deficits; KalSR^KO/KO^ mice evinced a decreased startle response in the pulse alone trials, demonstrating deficits in their baseline startle response. While KalGEF1^KO/KO^ mice showed reduced prepulse inhibition of acoustic startle relative to WT mice [25], neither the WT nor the KalGEF1^KO/KO^ mice showed the expected intensity dependence of prepulse induced inhibition [25]. The fact that both WT and KalSR^KO/KO^ mice showed a graded increase in inhibition of acoustic startle with increasing prepulse intensity supports the conclusion that ablation of *Kalrn* did not result in a hearing or response deficit. Several studies have noted significant co-morbidity of neuromuscular deficits and schizophrenia [80-82], which might be associated with deficits in *KALRN*.

## Competing interests

The authors declare that they have no competing interests.

## Authors’ contributions

Designed research: PM,BAE,REM, Performed research: PM,MY,LHC,XM,BAE,REM, Analyzed data: PM,LHC,JG,BAE,REM, Wrote the paper: PM,BAE,REM. All authors read and approved the final manuscript.
